# Variations in the fetal bovine serum and glucose concentration in the culture medium impact the viability of glioblastoma cells as evidenced through the modulation of cell cycle and reactive oxygen species: An *in vitro* study

**DOI:** 10.14440/jbm2025.0016

**Published:** 2025-08-28

**Authors:** Rimshia Naaz, Mahadevaswamy G. Kuruburu, Zonunsiami Leihang, Venugopal R. Bovilla, Rajalakshmi Rajashetty, Ramya C. Madhusetty, Vijaya Y. Vaagesh, SubbaRao V. Madhunapantula

**Affiliations:** 1 Center of Excellence in Molecular Biology and Regenerative Medicine, Department of Biochemistry, JSS Medical College, Faculty of Medicine, JSS Academy of Higher Education and Research, Mysuru, Karnataka 570015, India; 2 Department of Physiology, JSS Medical College, Faculty of Medicine, JSS Academy of Higher Education and Research, Mysuru, Karnataka 570015, India; 3 Special Interest Group in Cancer Biology and Cancer Stem Cells, JSS Medical College, Faculty of Medicine, JSS Academy of Higher Education and Research, Mysuru, Karnataka 570015, India

**Keywords:** Fetal bovine serum, Glucose, Reactive oxygen species, Rat glioblastoma cell line, C6 cell line, Human glioblastoma cell line, U-87 MG cell line

## Abstract

**Background:**

*In vitro* cell culture is essential for elucidating various signaling mechanisms and screening pharmacological agents to assess their safety and efficacy. However, cell proliferation and survival in culture can be significantly influenced by variations in the composition of the culture medium. For instance, variations in glucose and fetal bovine serum (FBS) concentrations can impact cell viability. Despite this, only a few studies have examined the impact of varied FBS and glucose concentrations in culture media on cell viability.

**Objective:**

This study investigated the mechanisms and cellular effects of glucose and FBS deprivation in glioblastoma cell lines.

**Methods:**

We systematically evaluated the impact of FBS and glucose deprivation on the proliferation and survival of rat C6 and human U-87 MG glioblastoma cell lines.

**Results:**

Glucose deprivation (0 mg/dL) significantly reduced the viability of C6 cells and moderately lowered the viability of U-87 MG cells, with partial recovery upon glucose supplementation (100 mg/dL, 400 mg/dL). Notably, FBS deprivation (0%) exerted a more profound effect, inducing the accumulation of reactive oxygen species and extensive cell death in both cell lines. Restoration of FBS (1, 2, 4, 6, 8, and 10%) recovered cell viability and reduced oxidative stress. Furthermore, both glucose and FBS deprivation altered antioxidant enzyme expression and mitochondrial function. Glucose and FBS deprivation also differentially affected protein kinase B phosphorylation, suggesting metabolic stress-induced signaling modulation.

**Conclusion:**

These findings highlight the differential responses of glioblastoma cells to glucose and FBS deprivation and underscore the importance of standardizing culture conditions, especially serum and glucose levels, when designing experiments involving glioblastoma cells.

## 1. Introduction

*In vitro* cell culture is fundamental for studying cellular and molecular mechanisms. It provides a platform to investigate the biology, physiology, biochemistry, and metabolism of both wild-type and diseased cells.^1^ Cells isolated from tissues are cultured in a nutrient-rich medium containing growth factors, glucose, proteins, salts, antibiotics, and other compounds.^2^ In addition, optimal pH, temperature, and humidity are maintained to support cell survival and proliferation.

Fetal bovine serum (FBS) is one of the most commonly used supplements in cell culture.^3^ FBS is rich in essential nutrients, such as amino acids, lipids, vitamins, minerals, hormones, and growth factors, which promote the growth, development, and proliferation of cells.^4^ These nutrients not only support various cellular processes, such as protein synthesis and energy production, but also fulfill the metabolic needs of cells. FBS enhances cell proliferation^5^ and provides support for cell adhesion and maintenance.^4^ Recent proteomic and metabolomic studies have identified over 1,800 proteins and more than 4,000 metabolites in FBS,^6^ which may serve critical functions in cell proliferation and survival through the activation of various signaling pathways.

Several studies have investigated the role of FBS in the *in vitro* system. Abramowicz *et al*.^7^ demonstrated that serum deprivation significantly affected the proliferation and viability of head and neck cancer cell lines.^7^ Similarly, serum deprivation has been reported to induce apoptosis and arrest breast cancer cells in the G0/G1 phase while downregulating the phosphatidylinositol 3-kinase (PI3K)/protein kinase B (Akt) signaling pathway through the upregulation upregulation of p53, p27, and B-cell lymphoma 2.^8^ Ni *et al*.^9^ showed that while serum deprivation could strongly induce the expression of damage-regulated autophagy modulator in liver cancer cell lines, serum starvation resulted in increased oxidative stress and reactive oxygen species (ROS) in prostate cancer cell lines.^10^ Despite these insights into the crucial role of FBS in cellular mechanisms, the exact pathways through which FBS exerts these effects remain unclear. Therefore, further exploration of the mechanisms by which FBS influences cellular processes is essential.

Another commonly used supplement in cell culture is glucose, a six-carbon sugar that serves as the primary energy source for cellular activities.^11^ Cells utilize glucose for energy production through aerobic glycolysis, known as the “Warburg effect.” Glucose concentrations in culture media typically range from 1 g/L to 10 g/L, depending on the requirements of different cells.^12^ Cancer cells, which have a high demand for glucose due to rapid proliferation, are particularly dependent on this energy source. As shown by Liu *et al.*,^13^ glucose deprivation leads to growth inhibition and triggers a cascade of cellular responses that induce cell cycle arrest. In one study, glucose withdrawal induced cell cycle arrest at the G0/G1 phase in hepatic cancer cells and the triple-negative breast cancer cell line MDA-MB-231.^14^ Another study by Huang *et al*.^15^ demonstrated that glucose deprivation induced apoptosis in A549, HI299, PC3, DU145, and U87-MG cells by modulating the PI3K/Akt/mechanistic target of rapamycin kinase pathways. While many studies confirmed that glucose starvation increases cancer cell death, others reported resistance to nutrient deprivation, leading to the discovery of glucose homeostasis mechanisms in cancer cells.^16^,^17^ Therefore, in this article, we aimed to explore the mechanisms and effects of glucose and FBS deprivation on glioblastoma cells.

## 2. Materials and methods

### 2.1. Materials

#### 2.1.1. Cell lines

U-87 MG (human glioblastoma) and C6 (rat glioblastoma) cell lines were procured from the National Centre for Cell Science (NCCS), Pune, Maharashtra, India. Human keratinocytes were obtained from the NCCS, Pune, India. Cell lines were characterized at regular intervals for their morphological similarity with the supplier’s data files and cultured under the influence of recommended media and antibiotics (10,000 U/mL penicillin and 10,000 μg/mL streptomycin).

#### 2.1.2. Reagents for cell culture

Dulbecco’s Modified Eagle’s Medium (DMEM) with high glucose (4.5 g/L) (Catalog number [Cat. no.] AL111), DMEM without glucose (Cat. no. AL186), Dulbecco’s phosphate-buffered saline (PBS; Cat. no. TL1006), and 0.25% trypsin-ethylenediaminetetraacetic acid (Cat. no. T001) were bought from HiMedia Laboratories Pvt. Ltd., India. FBS (Cat. no. 10270106), 500 mM GlutaMAX (Cat. no. 35050061), and 100× penicillin-streptomycin (Cat. no. 1540-122) were purchased from Thermo Fisher Scientific, USA.

#### 2.1.3. Plasticware

T25 (Cat. no. 156367) and T75 (Cat. no. 156499) cell culture flasks, Petri dishes sized 100 mm (Cat. no. 150464), 60 mm (Cat. no. 150462), and 30 mm (Cat. no. 150460), 5.0 mL (Cat. no. 170355) and 10 mL (Cat. no. 170356) serological pipettes, 96-well plates (Cat. no. 161093), and U-bottom plates (Cat. no. 163320) were from Thermo Fisher Scientific, USA. Microtips (10 μL, 200 μL, and 1,000 μL), microcentrifuge tubes sized 1.5 mL (Cat. no. 500010) and 2.0 mL (Cat. no. 500031), 1.8 mL cryovial tubes (Cat. no. 883192), 15 mL centrifuge tubes (Cat. no. 500041), and 50 mL centrifuge tubes (Cat. no. 941296) were from Tarsons Products Pvt. Ltd., India.

#### 2.1.4. Chemicals

Cholecalciferol (Vitamin D3; Cat. no. 67970), dimethyl sulfoxide (Cat. no. D8418), hybrimax dimethyl sulfoxide (Cat. no D2650), glucose-6-phosphate (G6P; Cat. no. G7250), glucose-6-phosphate-dehydrogenase (Cat. no. 74262), flavin adenine dinucleotide (Cat. no. F6625), nicotinamide adenine dinucleotide phosphate (Cat. no. N5755), 3-(4,5-dimethylthiazol-2-yl)-2,5-diphenyltetrazolium bromide (MTT; Cat. no. RM1131), dicoumarol (Cat. no. M1390), menadione (Cat. no. M5625), NP40 (Cat. no. 492016), protease inhibitor cocktail (Cat. no. S8820), ammonium persulfate (Cat. no. A3678), polyvinylidene fluoride membrane (Cat. no. IPVH00010), Coomassie blue G-250 (Cat. no. 64222), 2’,7’-dichlorodihydrofluorescein diacetate (H_2_DCFDA), sulforhodamine B (SRB; Cat. no. 230162), and JC-1 (Cat. no. T4069) came from Sigma Aldrich, USA.

Bovine serum albumin (Cat. no. 85171), radioimmunoprecipitation assay buffer (Cat. no. TCL131), polysorbate 20 (Tween 20; Cat. no. 23610), ethylenediaminetetraacetic acid (Cat. no. 40648), acrylamide (Cat. no. A8887), bis-acrylamide (Cat. no. MB005), N, N, N’, N’-tetramethylethylenediamine (Cat. no. T9281), glycine (Cat. no. 66327), tris buffer (Cat. no. 71033), sodium lauryl sulphate (Cat. no. 54468), dextrose (Cat. no. G7021), methanol (Cat. no. 65524), and trichloroacetic acid (Cat. no. 60677) were procured from SRL, India. Precision Protein StrepTactin-horseradish peroxidase conjugate (Cat. no. 1610380), Precision Plus Protein western blotting standard marker (Cat. no. 1610376), and enhanced chemiluminescence (ECL) substrate (Cat. no. 1705060) were procured from BioRad, USA.

#### 2.1.5. Kits

Bicinchoninic acid protein estimation kit (Cat. no. 23227) was purchased from Thermo Fisher Scientific, USA. High-throughput glutathione peroxidase (GPx) assay kit (Cat. no. 7512-100-K) was from R&D Biosystems, USA.

#### 2.1.6. Antibodies

Primary antibodies used in this study included: (i) pan-Akt (Cat. no. C67E7) from Cell Signaling Technology, USA, (ii) phosphorylated Akt (pAkt; Ser473; Cat. no. AP0140) from ABclonal, USA, (iii) nicotinamide adenine dinucleotide phosphate (NADPH) quinone oxidoreductase (NQO1; Cat. no. PAL969Hu01, PAL969Ra01), (iv) superoxide dismutase (Cat. no. PAB318Hu01 and PAB960Ra01), (v) glyceraldehyde 3-phosphate dehydrogenase (Cat. no. PAB932Hu02), (vi) beta-actin (Cat. no. CAB340Mi22), and (vii) GPx (Cat. no. PAA295Ra01, PAA295Hu01) from Cloudclone, USA, (viii) GPx (Cat.no. NBP1-33620) from Novus Biologicals, Centennial, USA, and (ix) α-Enolase (Cat. no. sc-271384) from Santacruz, USA. Horseradish peroxidase conjugated-secondary antibodies, anti-rabbit (Cat. no. sc-2357) and anti-mouse (Cat. no. sc2005), were procured from Santacruz, USA.

#### 2.1.7. Key instruments

The instruments used in this study included a biosafety cabinet (AC2-4S1; ESCO Lifesciences India Pvt. Ltd., India), a carbon dioxide incubator (Forma 371 Steri Cycle CO_2_ Incubator, Thermo Fisher Scientific, USA), a multimode plate reader (EnSpire2300, PerkinElmer Inc., USA), a fluorescence microscope (BX53, Olympus, Tokyo, Japan), a refrigerated centrifuge (5430R, Eppendorf, Germany), the VorTemp 56 (Labnet International Inc., USA), a probe sonicator (Vibracell-VCX500, Sonics & Materials Inc., USA), a gel rocker (Compact digital rocker, Thermo Fisher Scientific, China), the ChemiDoc system (Alliance Q9, UVITEC, UK), and a Leica Stellaris 5 Confocal microscope (Leica, Germany).

### 2.2. Determination of glucose impact on glioblastoma cell viability

**Figure 1:**
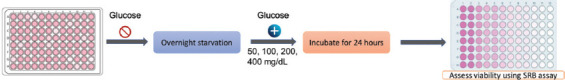
Schematic representation of the experimental workflow to assess the effect of glucose concentration on cell viability using the SRB assay. Cells were seeded into a 96-well plate and subjected to overnight glucose starvation. Following starvation, cells were treated with increasing concentrations of glucose (50, 100, 200, and 400 mg/dL) and incubated for 24 h. Cell viability was then assessed using the SRB assay. Abbreviation: SRB: Sulforhodamine B.

Rat C6 and human U-87 MG cells (1×10^4^ cells/well) were seeded into a 96-well plate in 100 μL complete medium (DMEM containing 4.5 g/L glucose, 10% FBS, and 5 mM glutaMAX, and 1% penicillin-streptomycin) and cultured in an incubator operating at 5% carbon dioxide, 95% relative humidity, and maintained at 37°C until the cells reached about 70% confluency. Subsequently, the existing medium was removed from the wells, and 100 μL of fresh medium without glucose and FBS was added to the flask. After 12 h of starvation, the medium was aspirated, and the cells were exposed to 100 μL of medium without glucose, or increasing concentrations of glucose at 50, 100, 200, and 400 mg/dL with 10% FBS. Cells were cultured for 24 h, and the viability was assessed using an SRB assay. Starved cells that continued to grow in a complete medium served as controls for comparison (Figure 1).

### 2.3. Assessment of FBS impact on glioblastoma cell viability

**Figure 2:**
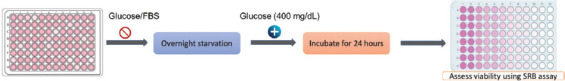
Schematic representation of the experimental workflow to assess the effect of fetal bovine serum (FBS) on cell viability using SRB assay. Cells were seeded into a 96-well plate and subjected to overnight glucose and FBS starvation. Following starvation, cells were treated with increasing concentrations of FBS (1,2,4,6,8, and 10%) in high glucose (400 mg/dL) and incubated for 24 h. Cell viability was then assessed using the SRB assay. Abbreviation: SRB: Sulforhodamine B.

A similar experimental setup (similar to Section 2.2) was used to assess the effect of FBS concentration on glioblastoma cells. C6 and U-87 MG cells were cultured as described earlier, and the medium was replaced with fresh medium without FBS. After 12 h of starvation, fresh medium (100 μL/well) containing increasing concentrations of FBS (1%, 2%, 4%, 6%, 8%, and 10%) and high glucose (400 mg/dL) was added to the culture. Cells were cultured for 24 h, and the viability was assessed using an SRB assay, as detailed in Figure 2. Cells that continued to grow in 10% FBS served as the control. Cells grown in 0% FBS after starvation were used as the no-FBS control.

### 2.4. Sulforhodamine B (SRB) assay

The SRB assay was performed as detailed by Skehan *et al*.^18^ Briefly, the control and treated cells were fixed with cold 50% trichloroacetic acid at 4°C for an hour. After incubation, the plate was gently washed under running tap water and air-dried. SRB solution (0.4% in 1% acetic acid) was added and the plates were incubated at room temperature for an hour. The plates were gently washed with 1% acetic acid to remove the excess unbound dye and air-dried to remove adhering moisture. Tris base (10 mM, pH ~10, 100 μL/well) was added and mixed to solubilize the protein-bound dye. The absorbance was measured at 510 nm, and cell viability was calculated using Equation I:

Cell viability (%) = (Absorbance of treated cells/Absorbance of untreated control) × 100 (I)

### 2.5. Measurement of ROS levels

Cellular ROS content was measured according to Shailasree *et al*.^19^ Briefly, the cells were cultured with glucose and FBS for 24 h as described in the previous sections. Then, 10 μM of H_2_DCFDA solution (10 μL/well) was added to the culture, and the cells were incubated for 30 min. After incubation, the cells were washed with PBS (200 μL × 2 washes), and 50 μL of PBS was added to each well before measuring the fluorescence intensity. The fluorescence intensity of dichlorofluorescein was measured on a multimode microplate reader programmed to operate at an excitation wavelength of 485 nm and an emission wavelength of 535 nm. Fold change in relative fluorescence units was compared to that in the cells that were grown in the complete medium, with data presented as a bar graph.

### 2.6. Measurement of nicotinamide adenine dinucleotide phosphate quinone oxidoreductase activity

Nicotinamide adenine dinucleotide phosphate quinone oxidoreductase activity was measured by quantifying the NADPH produced upon G6P oxidation by G6P dehydrogenase. The NADPH quantity was estimated in terms of the amount of menadiol produced through the reduction of menadione as detailed by Prochaska and Santamaria.^20^ The formation of blue-colored formazan was measured at 610 nm on a multimode plate reader. To measure the background activity contributed by other reductases, dicoumarol (a specific inhibitor of NQO1) was used. Aliquots of total protein (10 μg/40 μL) were incubated with 200 μL of NQO1 cocktail with and without dicoumarol (Table 1).

**Table 1:** Preparation of a solution cocktail with and without dicoumarol

Reagent	Volume	

	With dicoumarol	Without dicoumarol
Tris buffer (pH 7.5)	50 μL	50 μL
Bovine serum albumin (2%)	33 μL	33 μL
Tween 20 (1.5%)	6.6 μL	6.6 μL
Glucose-6-phosphate (150 mM)	6.6 μL	6.6 μL
Flavin adenine dinucleotide (7.5 mM)	0.66 μL	0.66 μL
Nicotinamide adenine dinucleotide phosphate (5 mM)	0.6 μL	0.6 μL
Glucose-6-phosphate dehydrogenase (2 units/μL)	1 μL	1 μL
3-(4,5-dimethylthiazol-2-yl)- 2,5-diphenyltetrazolium bromide (1%)	30 μL	30 μL
Menadione	0.66 μL	0.66 μL
Dicoumarol (10 mM)	2 μL	-
Water	868.88 μL	870.88 μL

The absorbance was measured at 610 nm for 30 min at 1-min intervals. NQO1 activity was calculated by subtracting the readings of samples containing the inhibitor from the ones without. Following this, the optical density value per minute was calculated, and mole units were determined by multiplying the optical density/min/molar extinction coefficient of MTT (11,300 M/cm) with the protein concentration of a sample.^20^ The NQO1 activity was expressed as μmol/min/mg protein.

### 2.7. Measurement of cell death by dual staining with acridine orange (ao) and ethidium bromide

To determine cell death-related changes, an acridine orange and ethidium bromide staining protocol was used.^21^ After treatment, the control and treated cells (0.5 × 10^6^) were trypsinized and mixed to create a single-cell suspension. The trypsinized cells were neutralized by adding complete media. The cell suspension was centrifuged for 5 min at 900 × g at room temperature. The cell pellet was washed once with 500 μL of PBS and centrifuged for 5 min at 900 × g. Following this, the PBS was completely removed. Ethidium bromide and acridine orange mixture (100 μg/mL in a 1:1 ratio) was added to the cell pellet and incubated for 10 min at room temperature. The stained cells were examined under a fluorescence microscope (BX 53, Olympus Corporation, Japan) at 20 × magnifications, operating with tetramethylrhodamine and fluorescein isothiocyanate filters. The percentage of dying cells relative to the total cells/field was calculated and presented as a bar graph.

### 2.8. Analysis of cell cycle using a 2-step 4’,6-diamidino-2-phenylindole staining procedure

To determine the changes in cell cycle stages upon treatment with glucose or FBS, a cell cycle analysis was carried out according to the protocol by Chikkegowda *et al*.^22^ Briefly, about 1 × 10^6^ cells from the control and experimental groups were harvested following trypsinization and centrifuged at 500 × g for 5 min. The cell pellet was washed twice with PBS (500 μL), and the cells were then fixed with ice-cold 70% ethanol by dropwise addition of the cell suspension into the alcohol. The fixation was conducted overnight at 4°C. The fixed cells were then centrifuged and washed twice with 500 μL of PBS to remove any traces of ethanol. 4’,6-diamidino-2-phenylindole (DAPI) staining solution (1.0 μg/mL DAPI and 0.1% Triton X-100 in PBS) was added to the cell pellet and left to incubate for 5–7 min. The cell suspension with DAPI was loaded onto the NC-Slide 8 (ChemoMetec, Denmark), and DAPI-stained cells were detected and quantified by using the NucleoCounter^®^ NC-3000 system (ChemoMetec, Denmark).

### 2.9. Measurement of glutathione (GSH) levels

The total GSH level was measured according to Rahman *et al.*,^23^ using the enzymatic recycling method. Briefly, aliquots of 10 μg of total protein were loaded into a 96-well microtiter U-bottom plate and incubated with 60 μL of NADPH solution (0.89 mM) for 60 s. An equal volume (1:1 ratio) of 5,5’-dithiobisnitrobenzoic acid (DTNB; 1.6 mM) and GSH reductase (3.3 units/mL), yielding a combined volume of 120 μL, was added to each well. Measurements were taken at an absorbance of 412 nm. The absorption was monitored for 4 min at 1-min intervals. The concentration of total GSH was determined by comparing the colorimetric rate of DTNB reduction with that of a known amount of reduced GSH. The assay is based on the reaction of GSH with DTNB (also known as Ellman’s reagent) that produces the 5-thionitrobenzoic acid (TNB) chromophore, which has a maximal absorbance at 412 nm, and oxidized GSH-TNB adducts. The rate of change in absorbance (at 412 nm at each minute) was found to be directly proportional to the total concentration of GSH. The concentration of GSH in the unknown sample was determined through calculations using the linear equation or the regression curve generated from several standards of GSH (0.5–32.5 μM). Results were expressed as the nmol of total GSH per mg of protein.

### 2.10. Measurement of GPx activity

The GPx activity was determined using a commercially available high-throughput kit from R&D Systems (Cat No. 7512-100-K). Briefly, 10 μg of total protein was loaded into a 96-well microtiter U-bottom plate along with 140 μL of assay buffer, 20 μL of reaction mixture, and 20 μL of GPx, which served as a positive control. To initiate the reaction, 20 μL of cumene hydrogen peroxide was added, and the plate was read immediately at 340 nm every 10 s for 5 min. Data were analyzed using the formula in Equation II:

GPx activity = ΔA340/min/0.00379 μM−1 × 0.2 mL × sample dilution/mL (II)

### 2.11. Measurement of superoxide dismutase activity

Superoxide dismutase (SOD) activity in the serum was determined using a high-throughput SOD assay kit from R&D Systems (Cat No: 7501-500-K). Briefly, 25 μL of serially diluted standards and samples (10 μg) were loaded into a 96-well plate. Following this, 150 μL of master mix containing 10× SOD buffer, water-soluble tetrazolium-1 reagent, xanthine oxidase, and distilled water was added to the wells. To initiate the reaction, 25 μL of 1× xanthine solution was added and immediately read at 450 nm every minute for 10 min at room temperature on a multimode plate reader (EnSpire2300, PerkinElmer Inc., USA). The slope of the curve was obtained by plotting absorbance at 450 nm on the Y-axis and time on the X-axis. The percentage inhibition was calculated using the formula in Equation III:


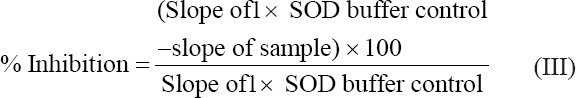


One unit of SOD activity is defined as the amount of enzyme required to inhibit 50% of water-soluble tetrazolium-1 formazan formation. The protein concentration required to achieve this level of inhibition was calculated based on protein estimation. Specific activity was estimated by dividing one unit of SOD by the corresponding amount of protein and expressed as U/mL.

### 2.12. Determination of mitochondrial membrane potential

Briefly, 2 × 10^5^ cells were seeded into a 30 mm dish and cultured as aforementioned. The control and experimental cells were stained with JC-1 dye (added at 5 μg/mL) by incubating at 37°C for 30 min. After 30 min, the cells were washed with PBS 3 times at an interval of 5 min each. Following this step, the cells were mounted on a clean microscopic slide and observed under a confocal microscope (Leica Stellaris 5, Leica, Wetzlar, Germany). A magnification of 40× was used to capture the images. A minimum of 4–6 fields/slide were captured and calculation was performed by quantifying the images using Image J software version 8.0.

### 2.13. Western blot analysis

The protein lysates were harvested from the cells using the radioimmunoprecipitation assay lysis buffer containing 50 mM of 4-(2-hydroxyethyl)-1-piperazineethanesulfonic acid (pH 7.5), 150 mM of sodium chloride, 10 mM of ethylenediaminetetraacetic acid, 10% glycerol, 1% Triton X-100, 1 mM of sodium orthovanadate, 0.1 mM of sodium molybdate, and 1 mM of phenylmethylsulphonyl fluoride, according to a procedure described previously.^24^ Protein content was estimated by using the bicinchoninic acid method with bovine serum albumin as standards. The electrophoresis was performed by loading 50 μg total protein per lane into 12% sodium dodecyl sulfate polyacrylamide gels. The proteins were separated by electrophoresis at 60V for 3 h. The separated proteins were then transferred to the polyvinylidene fluoride membrane as detailed in a prior study.^25^ Following this transfer, blots were blocked with 5% skim milk in Tris-buffered saline supplemented with Tween 20 (10 mM Tris-hydrochloric acid [pH 7.6], 150 mM sodium chloride, 0.5% Tween 20). The blots were incubated in the primary antibodies (Table 2) for about 4 h on a shaker at room temperature. Post-incubation, the blots were washed with Tris-buffered saline supplemented with Tween 20 (3 times with a 10-min interval). Subsequently, the blots were incubated with horseradish peroxidase-conjugated secondary antibodies (anti-rabbit; 1:10,000 dilution) for about 2 h on a shaker at room temperature. The protein bands were visualized by adding ECL onto the blots and visualized on the ChemiDoc system. The intensity of protein bands was quantitatively determined by using Image-J software version 8.0 and represented as bar graphs.

**Table 2:** Dilution of primary antibodies

Antibody	Dilution
Phosphorylated protein kinase B (Ser 473)	1:1,000
Total protein kinase B	1:1,000
Nicotinamide adenine dinucleotide phosphate quinone oxidoreductase	1:1,000
Superoxide dismutase	1:1,000
Glutathione peroxidase	1:1,500
Beta-actin	1:1,000

### 2.14. Statistical analysis

All results were presented as the mean of two independent experiments ± standard error of the mean (SEM). GraphPad Prism version 8.0 (GraphPad Software, USA) was used for statistical analyses. The results were subjected to one-way analysis of variance to compare differences between control and test groups. Tukey’s *post hoc* test was used as indicated, and *p*<0.05 was considered statistically significant.

## 3. Results

### 3.1. Effect of glucose on U-87 MG and C6 cell viability

Glucose is the cells’ main source of energy and nutrients; hence, it is essential for cell growth and proliferation. The glioblastoma cell lines U-87 MG and C6 cells were cultured in a medium containing 450 mg/dL of glucose. However, the impact of high glucose (450 mg/dL) on cell survival, proliferation rates, and death is unknown. Recent studies have shown the induction of ROS when cells were cultured in a hyperglycemic medium (450 mg/dL). Hence, we hypothesized that the hyperglycemic concentration of glucose in cell culture medium may influence the proliferation and survival rates of glioblastoma cells. Therefore, to determine the impact of glucose on cell viability, glioblastoma C6 and U-87 MG cells were cultured in media supplemented with rising concentrations of glucose, and cell viability was measured accordingly. In addition, intracellular ROS levels were measured using the H_2_DCFDA protocol (as detailed in Section 2.5).

**Figure 3:**
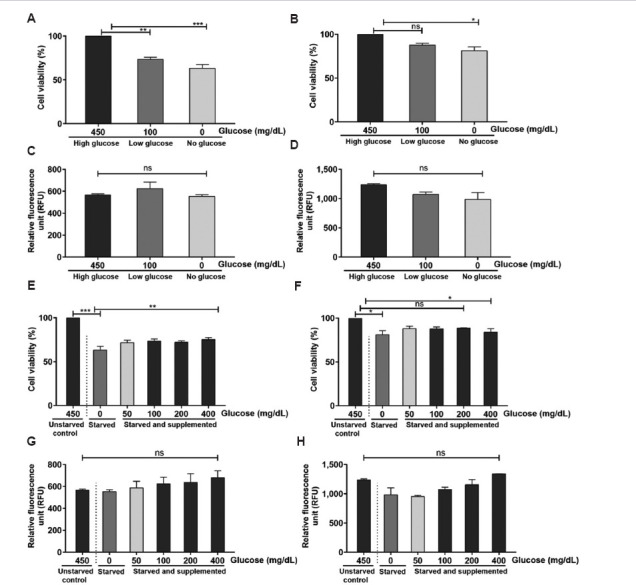
Glucose deprivation impacted cell viability and intracellular reactive oxygen species (ROS) levels in glioblastoma cells. Viability of (A) C6 cells and (B) U-87 MG cells cultured in media with no glucose and low glucose compared to high glucose. Intracellular ROS levels of (C) C6 cells and (D) U-87 MG cells in media with no glucose and low glucose compared to high glucose. Viability of (E) C6 cells and (F) U-87 MG cells cultured in medium supplemented with increasing concentrations of glucose (0, 50, 100, 200, and 400 mg/dL) compared to the control. Intracellular ROS levels in (G) C6 cells and (H) U-87 MG cells cultured in medium with varying concentrations of glucose (0, 50, 100, 200, and 400 mg/dL) compared to the control. Viability was assessed using a sulforhodamine B assay, and ROS was measured using 2’,7’-dichlorodihydrofluorescein diacetate dye. The data represent the mean of two independent experimental values with at least three replicate wells in each experiment. The statistical analysis was conducted using Tukey’s *post hoc* test. Statistical significance was determined at *p<0.05, **p<0.01, and ***p<0.001. ns refers to not significant No glucose is 0 mg/dL, low glucose is 100 mg/dL, and high glucose is 450 mg/dL.

Briefly, the glioblastoma cells were cultured in media supplemented with high glucose (450 mg/dL, hyperglycemic state), low glucose (100 mg/dL, normal glycemic state), and without glucose (0 mg/dL, a glycemic state) for 24 h, and the viability was determined using an SRB assay. Data analysis showed that C6 cells cultured in high-glucose media exhibited the highest viability, set as 100%. In comparison, cells cultured in low-glucose media showed reduced viability at 73% relative to the high-glucose group, while those in glucose-free media demonstrated further reduced viability at 63% (Figure 3A). In contrast, the U-87 MG cells showed no statistically significant changes in cell viability in low-glucose media (87% viability) (Figure 3B). However, in glucose-free media, the U-87 MG cells showed a significant 21% decrease in cell viability compared to their counterparts cultured in high-glucose media (Figure 3B). Notably, the evaluation of intracellular ROS levels showed no significant changes in both glioblastoma cell lines (Figure 3C and D). Subsequently, the viability of C6 and U-87 MG cells cultured in varying glucose concentrations was tested to identify the optimal glucose concentration in the medium. The C6 cells cultured in glucose-free media showed reduced viability by about 63%. When glucose was supplemented, viability increased to 71% at 50 mg/dL, 73% at 100 mg/dL, 72% at 200 mg/dL, and 75% at 400 mg/dL (Figure 3E). This increase in viability was accompanied by a slight increase in intracellular ROS levels, although the changes were not statistically significant (Figure 3F). In U-87 MG cells, no significant difference in viability was observed across the glucose concentrations tested (Figure 3G). However, a slight decrease in viability was noted at 0 mg/dL (81% viability compared to the control cells growing in 450 mg/dL glucose medium) (Figure 3G). Furthermore, the analysis of intracellular ROS levels revealed no significant changes across the groups (Figure 3H). In summary, the absence of glucose in the culture medium reduced the viability of cells, but supplementation with glucose partially restored cell viability.

### 3.2. Activation of protein kinase B signaling in glioblastoma cells under glucose deprivation

**Figure 4:**
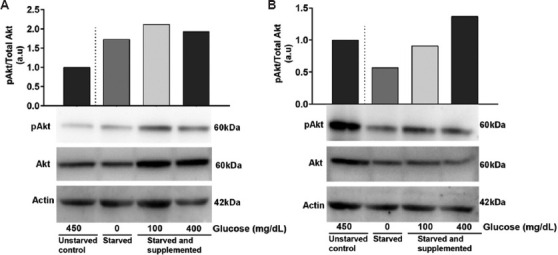
Impacts of varying concentrations of glucose on the expression of phosphorylated protein kinase B (pAkt) in glioblastoma cells. Glioblastoma cells were cultured in media supplemented with increasing concentrations of glucose (0 mg/dL, 100 mg/dL, and 400 mg/dL) for 24 h, and protein lysates were subjected to Western blotting to assess the expression of pAkt, total Akt, and internal control beta-actin. (A) Representative blots showing the expression of p-Akt, total Akt, and beta-actin in C6 cells (below) and graph representing C6 cells cultured in media with no glucose (0 mg/dL), low glucose (100 mg/dL) and high glucose (400 mg/dL) compared to cells cultured in 450 mg/dL glucose-containing medium (above). (B) Representative blots showing the expression of p-Akt, total Akt, and beta-actin in U-87 MG cells (below) and graph representing U-87 MG cells cultured in media with no glucose (0 mg/dL), low glucose (100 mg/dL) and high glucose (400 mg/dL) compared to the cells cultured in medium containing 450 mg/dL glucose (above). All Western blotting experiments were performed once.

The expression levels of pAkt and total Akt were assessed to elucidate the mechanisms underlying glucose’s influence on glioblastoma cells. Western blot analysis revealed increased expression of pAkt in C6 cells cultured in 0 mg/dL, 100 mg/dL, and 400 mg/dL compared to the control (450 mg/dL) (Figure 4A). In U-87 MG cells, a visible decrease in pAkt expression was observed at 0 mg/dL. However, a moderate and substantial increase in pAkt was noted at 100 mg/dL and 400 mg/dL compared to the control cells cultured in 450 mg/dL glucose-supplemented medium (Figure 4B).

### 3.3. Antioxidant responses and mitochondrial membrane potential under normal and high glucose conditions

Intracellular ROS levels are controlled by a fine balance between the processes that produce and destroy these unstable and highly reactive radicals.^26^ Higher ROS levels in cells are primarily due to either lowered GSH or NADPH, a decrease in the redox enzymes, such as NQO1, GPx, SOD, catalase, and peroxidase, or a combination of these two mechanisms. Hence, we investigated the impact of glucose concentration on the expression and activity levels of antioxidant enzymes, intracellular GSH content, and mitochondrial membrane integrity in the glioblastoma cells.

**Figure 5:**
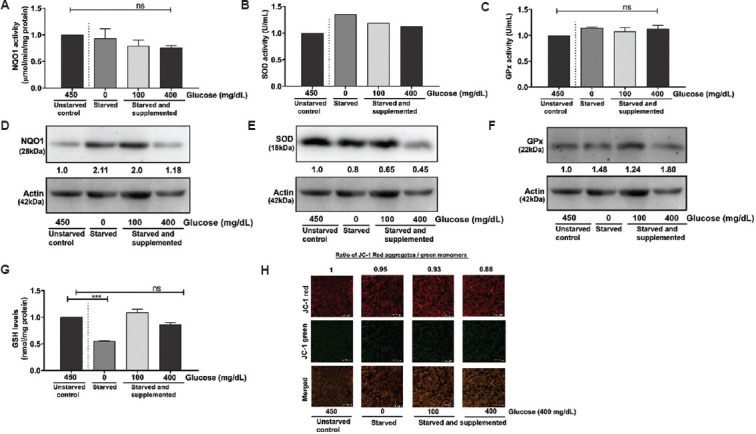
Variations in the antioxidant enzyme activity levels and expression, glutathione content, and mitochondrial membrane potential in C6 cells. Glioblastoma cells were starved and supplemented with different glucose concentrations (0 mg/dL, 100 mg/dL, and 400 mg/dL). The expression and activity of antioxidant enzymes and stability in mitochondrial membrane potential were assessed. (A) NQO1, (B) SOD, and (C) GPx activity in C6 cells in 0 mg/dL glucose (starved), 100 mg/dL, and 400 mg/dL glucose (starved and supplemented) conditions compared to 450 mg/dL glucose (unstarved) condition. Representative quantified images of blots depicting (D) NQO1 expression, (E) SOD expression, (F) GPx expression, and (G) GSH levels in 0 mg/dL glucose (starved), 100 mg/dL, and 400 mg/dL glucose (starved and supplemented) conditions compared to 450 mg/dL glucose (unstarved) condition. (H) Representative quantified images of JC-1 dye for mitochondrial membrane potential expression in 0 mg/dL glucose (starved), 100 mg/dL, and 400 mg/dL glucose (starved and supplemented) compared to 450 mg/dL glucose (unstarved). Scale bar: 72.7 μm, magnification: 20×. The data represent the mean of two independent experimental values with at least three replicate wells in each experiment. The statistical analysis was conducted using Tukey’s *post hoc* test. Statistical significance was determined at ***p<0.001. ns refers to not significant. Abbreviations: GPx: Glutathione peroxidase; GSH: Glutathione; NQO1: Nicotinamide adenine dinucleotide phosphate quinone oxidoreductase; SOD: Superoxide dismutase.

Data analysis of C6 cells revealed that glucose deprivation resulted in a non-significant increase in NQO1 activity, which decreased upon supplementation with 100 mg/dL and 400 mg/dL of glucose (Figure 5A). The expression of NQO1 showed an increase at 0 mg/dL and 100 mg/dL but was reduced at 400 mg/dL (Figure 5D). GPx activity showed no changes (Figure 5C), while its expression was increased across all glucose concentrations (Figure 5F). While there was an increase in SOD activity compared to the non-glucose-deprived control (Figure 5B), SOD expression dose-dependently decreased with increasing glucose concentrations (Figure 5E). GSH levels were reduced at 0 mg/dL but showed a moderate increase at 100 mg/dL and 400 mg/dL (Figure 5G). The mitochondrial membrane potential was marginally modified at 400 mg/dL (Figure 5H).

**Figure 6:**
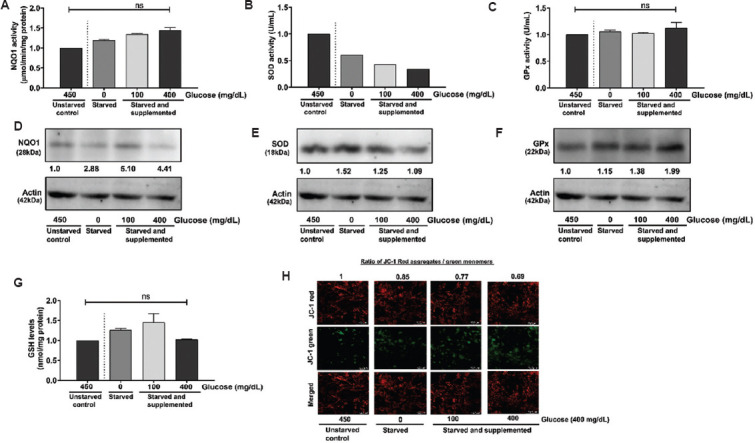
Variations in the antioxidant enzyme activity levels and expression, glutathione content, and changes in mitochondrial membrane potential in U-87 MG cells. Glioblastoma cells were starved and supplemented with different glucose concentrations (0 mg/dL, 100 mg/dL, and 400 mg/dL). The expression and activity of antioxidant enzymes and stability in mitochondrial membrane potential were assessed. (A) NQO1, (B) SOD, and (C) GPx activity in U-87 MG cells under 0 mg/dL glucose (starved), 100 mg/dL, and 400 mg/dL glucose (starved and supplemented) conditions compared to 450 mg/dL glucose (unstarved) condition. Representative quantified images of blots depicting (D) NQO1 expression, (E) SOD expression, (F) GPx expression, and (G) GSH levels under 0 mg/dL glucose (starved), 100 mg/dL, and 400 mg/dL glucose (starved and supplemented) conditions compared to 450 mg/dL glucose (unstarved) condition. (H) Representative quantified images of JC-1 dye for mitochondrial membrane potential expression in 0 mg/dL glucose (starved), 100 mg/dL, and 400 mg/dL glucose (starved and supplemented) compared to 450 mg/dL glucose (unstarved). Scale bar: 72.2 μm, magnification: 20×. The data represent the mean of two independent experimental values with at least three replicate wells in each experiment. The statistical analysis was conducted using Tukey’s post hoc test. ns refers to not significant. Abbreviations: GPx: Glutathione peroxidase; GSH: Glutathione; NQO1: Nicotinamide adenine dinucleotide phosphate quinone oxidoreductase; SOD: Superoxide dismutase.

In U-87 MG cells, glucose deprivation increased the activity and expression of NQO1 (Figure 6A). Supplementation of the medium with glucose further elevated the expression of NQO1 (Figure 6D). Similarly, GPx activity and expression also increased (Figure 6C and F). In contrast, the activity of SOD was reduced at 0 mg/dL and at varying concentrations of glucose supplementation (Figure 6B), while the expression of SOD was elevated across all glucose conditions (Figure 6E). GSH levels increased non-significantly at 0 mg/dL and 100 mg/dL and returned to normal control levels at 400 mg/dL (Figure 6G). The mitochondrial membrane potential showed a moderate decrease at 400 mg/dL (Figure 6H).

### 3.4. Reversal of starvation-induced G0/G1 cell cycle arrest through glucose supplementation

**Figure 7:**
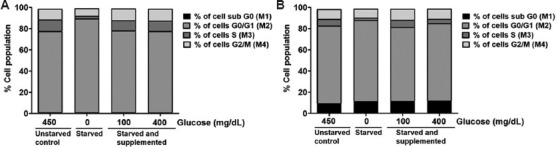
Glucose-deprivation-induced G0/G1 arrest is reversed through glucose supplementation. Representative bar graph depicting the changes in cell cycle stages of (A) C6 cells and (B) U-87 MG cells cultured in 0 mg/dL glucose (starved), 100 mg/dL glucose, and 400 mg/dL glucose compared to 450 mg/dL glucose (unstarved).

Exponentially growing C6 and U-87 MG cells were starved of glucose overnight and subsequently exposed to 100 mg/dL and 400 mg/dL of glucose-containing media for 24 h to determine if glucose supplementation could reverse the starvation-induced G0/G1 cell cycle arrest. Following the growth, the cells were stained with DAPI and analyzed using the Nucleocounter-3000 system. Data analysis revealed that glucose withdrawal induced cell cycle arrest in the G0/G1 phase. Supplementation of glucose reversed this effect, as evidenced by a decrease in G0/G1 cell population (similar in unstarved cells) in both C6 and U-87 MG glioblastoma cells (Figure 7A and B).

### 3.5. FBS is essential for the survival of glioblastoma cells

**Figure 8:**
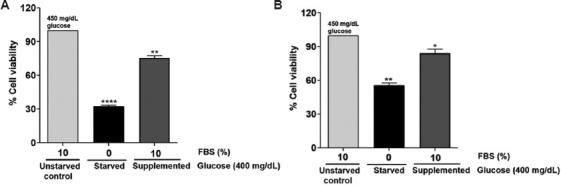
Deprivation of fetal bovine serum (FBS) affects the viability of glioblastoma cells. Glioblastoma cells were supplemented and starved with FBS for 24 h. Representative bar graph showing changes in cell viability and growth of (A) C6 cells and (B) U-87 MG cells in 10% FBS, 0% FBS, compared to unstarved 10% FBS in high glucose (450 mg/dL) at 24 h. The values are expressed as mean ± standard error of the means of two independent experimental values. The statistical analysis was conducted using Tukey’s *post hoc* test. Statistical significance determined at *p<0.05, **p<0.01, and ****p<0.0001. ns refers to not significant.

Cell culture medium lacking FBS has been widely used in cell starvation and synchronization.^27^ The duration of serum starvation varies between cell lines, and continuous serum starvation may cause cell death. Therefore, it is important to determine the impact of serum starvation on cell viability. In this study, we investigated the effect of serum deprivation on the growth and viability of glioblastoma cells. Experimentally, the cells were deprived of serum for about 12 h and subsequently cultured in medium supplemented with increasing concentrations of FBS or not supplemented with FBS as detailed in Section 2.3. Removal of FBS from culture media significantly reduced the number of cells. However, supplementation of FBS to the serum-starved cells partially restored cell viability. Data analysis revealed that, in the absence of FBS, C6 cells exhibited a viability of 30% (Figure 8A), while U-87 MG cells showed a viability of 50% (Figure 8B), compared to unstarved cells that maintained 100% viability. Notably, when supplemented with 10% FBS, cell viability increased to approximately 75% in C6 cells and 80% in U-87 MG cells. Thus, this experiment highlights the crucial role of FBS in supporting the survival of glioblastoma cells.

### 3.6. Serum deprivation-induced cell death without cytostasis in glioblastoma cells

**Figure 9:**
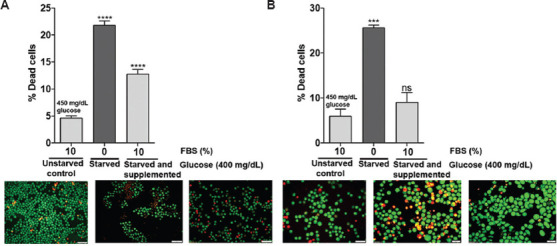
Deprivation of serum triggered the death of glioblastoma cells. Glioblastoma cells that were starved and supplemented with 10% fetal bovine serum (FBS) in high glucose conditions (400 mg/dL) were tested for apoptosis using the acridine orange and ethidium bromide staining method. Representative bar graph in (A) C6 cells showing the percentage of dead cells with 0% FBS (starved) and 10% FBS (supplemented) in high glucose condition (400 mg/dL) compared to 450 mg/dL (unstarved) at 24 h (above). Image representing the number of dead cells stained red and viable cells stained green (below) for (A) C6 cells and (B) U-87 MG cells. Scale bar: 50 μm, magnification: 20×. The values are presented as the mean ± standard error of the means of four different sections. The statistical analysis was conducted using Tukey’s *post hoc* test. Statistical significance was determined at ***p<0.001 and ****p<0.0001. ns refers to not significant.

Given the observed reduction in viability in serum-deprived glioblastoma cells, we evaluated whether the effect was attributable to cell death or cytostasis. FBS-deprived cells showed significantly increased levels of dying cells in U-87 MG and C6 glioblastoma cells (Figure 9A and B), thus suggesting that FBS deprivation triggers cytocidal processes.

### 3.7. Impact of serum reduction on cell viability and ROS levels

**Figure 10:**
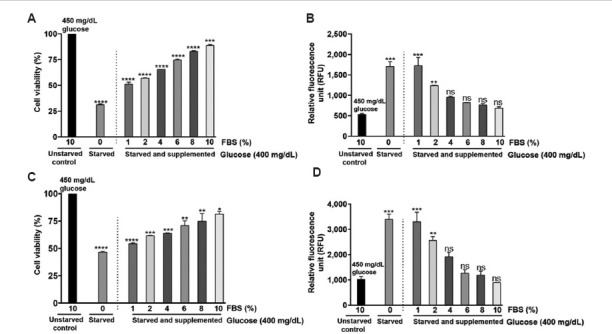
Fetal bovine serum (FBS) influences the generation of intracellular reactive oxygen species (ROS) and cell viability in glioblastoma cells. Cultures grown in complete media (4.5 g/L) were starved to synchronize the cells, and then the cells were treated with different concentrations of FBS. Post-treatment, cell viability and ROS levels were measured at 24 h. Percentage of cell viability of (A) C6 cells and (C) U-87 MG cells cultured with 0% FBS (starved) and increasing concentration of FBS (1–10%) (starved and supplemented) compared to unstarved control (450 mg/dL with 10% FBS) at 24 h. Intracellular ROS levels of (B) C6 cells and (D) U-87 MG cells cultured with 0% FBS (starved) and increasing concentration of FBS (1–10%) (starved and supplemented) compared to unstarved control (450 mg/dL with 10% FBS) at 24 h. The values are represented as mean ± standard error of the means of two independent experimental values. The statistical analysis was conducted using Tukey’s *post hoc* test. Statistical significance determined at *p<0.05, **p<0.01, ***p<0.001 and ****p<0.0001. ns refers to not significant.

In the previous experiments, we observed a significant decrease in cell viability when cultured in a serum-free medium. The observed decline in cell count is attributable to elevated cytocidal damage. To further investigate the mechanisms causing cell death, we measured ROS levels using the H_2_DCFDA protocol as detailed by Shailasree *et al*.^19^ Procedurally, glioblastoma cells were cultured in a medium containing different concentrations of FBS (1–10%), and after 24 h, the viability was measured using an SRB assay. Simultaneously, the generation of ROS was assessed using H_2_DCFDA. Data analysis revealed a dose-dependent increase in the viability of C6 cells with increasing FBS concentration (Figure 10B). Furthermore, an increase in FBS concentration decreased ROS levels (Figure 10A). In U-87 MG cells, the viability showed a dose-dependent response with varying FBS concentrations (Figure 10D). A noticeable decrease in ROS levels was observed with increasing FBS concentrations (Figure 10C).

### 3.8. Modulation of expression/activity of antioxidant enzymes and mitochondrial membrane potential upon stimulation with FBS

To demonstrate the impact of FBS on cellular oxidative stress, cells were starved of glucose and FBS. Following starvation, they were supplemented with varying concentrations of FBS (1%, 2%, 4%, 6%, 8%, and 10%) under a high glucose condition (400 mg/dL) and the data were compared with cells growing in complete media containing 450 mg/dL glucose with 10% FBS (*i*.*e*., unstarved).

**Figure 11:**
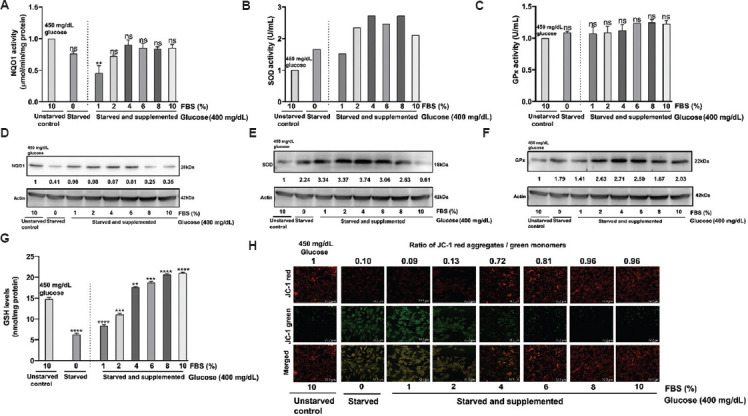
Impact of fetal bovine serum (FBS) on the mitochondrial membrane potential and antioxidant enzymes activities and expressions in C6 cells. The cells were grown in complete media (4.5 g/L) and were then starved of FBS to synchronize the cultures. Subsequently, the cells were allowed to grow in a medium supplemented with different concentrations of FBS (0–10%) for 24 h. (A) NQO1 activity, (B) SOD activity, and (C) GPx activity in C6 cells cultured with 0% FBS (starved), 1–10% (starved and supplemented) compared to the cells cultured in a medium containing 450 mg/dL glucose in 10% FBS (unstarved). Representative quantified blots showing the expression of (D) NQO1, (E) SOD, and (F) GPx in C6 cells grown in 0% FBS (starved), 1–10% compared to the cells cultured in a medium supplemented with 450 mg/dL glucose and 10% FBS (unstarved). (G) GSH levels in C6 cells. (H) Representative photomicrographs of JC-1 dye-stained cells depicting the variations in mitochondrial membrane potential in C6 cells. Scale bar: 72.2 μm, magnification: 20×. The data represent the mean of two independent experimental values with at least three replicate wells in each experiment. The statistical analysis was conducted using Tukey’s *post hoc* test. Statistical significance was determined at *p<0.05, **p<0.01, and ***p<0.001. ns refers to not significant. Abbreviations: GPx: Glutathione peroxidase; GSH: Glutathione; NQO1: Nicotinamide adenine dinucleotide phosphate quinone oxidoreductase; SOD: Superoxide dismutase.

Data analysis of C6 cells showed that serum deprivation resulted in a non-significant decrease in NQO1 activity, which further declined when the cells were cultured in a 0% FBS medium. However, increasing FBS from 2% to 10% did not affect the activity of NQO1(Figure 11A). NQO1 expression followed a similar pattern with no changes in expression (Figure 11D). In contrast, SOD activity increased upon serum deprivation, and further addition of FBS led to a rise in SOD activity (Figure 11B), accompanied by higher SOD expression levels (Figure 11E). No changes were observed in GPx activity across the groups (Figure 11C), but an increase in GPx expression was noted (Figure 11F). GSH levels were reduced in the 0% FBS group, but a dose-dependent increase was seen with higher FBS concentrations (Figure 11G). In addition, C6 cells displayed a significant green monomeric signal in serum-depleted cells, indicating elevated ROS levels. The addition of FBS to the culture medium restored membrane potential, as evidenced by the appearance of red J-aggregates in these cells (Figure 11H).

**Figure 12:**
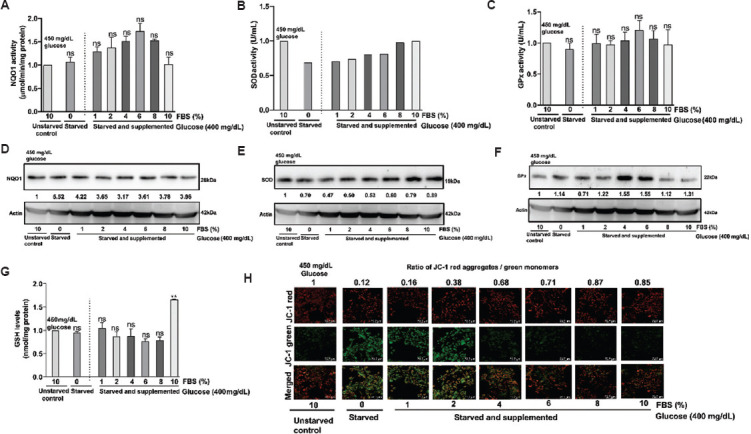
Impact of fetal bovine serum (FBS) on the mitochondrial membrane potential and antioxidant enzymes activities and expressions in U-87 MG cells. The cultures were grown in complete medium supplemented with 4.5 g/L glucose and 10% FBS. Subsequently, the cells were starved and cultured in medium containing different concentrations of FBS (0–10%) for 24 h. (A) NQO1 activity, (B) SOD activity, and (C) GPx activity, in U-87 MG cells cultured with 0% FBS (starved), 1–10% (starved and supplemented) compared to the cells cultured in a medium containing 450 mg/dL glucose in 10% FBS (unstarved). Representative blots showing the expression of (D) NQO1, (E) SOD, and (F) GPx in U-87 MG cells cultured with 0% FBS (starved), 1–10% (starved and supplemented) compared to the cells cultured in a medium containing 450 mg/dL glucose in 10% FBS (unstarved). (G) Cellular GSH level. (H) Representative photomicrographs of cells stained with JC-1 dye for measuring the mitochondrial membrane potential. Scale bar: 72.2 μm, magnification: 20×. The data represent the mean of two independent experimental values with at least three replicate wells in each experiment. The statistical analysis was conducted using Tukey’s *post hoc* test. Statistical significance was determined at *p<0.05, **p<0.01, and ***p<0.001. ns refers to not significant. Abbreviations: GPx: Glutathione peroxidase; GSH: Glutathione; NQO1: Nicotinamide adenine dinucleotide phosphate quinone oxidoreductase; SOD: Superoxide dismutase.

Data analysis of U-87 MG cells revealed no changes in NQO1 activity upon serum deprivation, but FBS supplementation led to a non-significant increase in NQO1 activity (Figure 12A). NQO1 expression exhibited an increase in 0–8% FBS, which then decreased with the addition of 10% FBS (Figure 12D). In contrast, SOD activity increased with serum deprivation, and further addition of FBS resulted in an elevation in SOD activity (Figure 12B). However, SOD expression stayed unchanged (Figure 12E). No changes in GPx activity were observed across the groups (Figure 12C), but there was an increase in GPx expression (Figure 12F). GSH levels remained consistent across most groups, except in the 10% FBS group (Figure 12G). Similar to C6 cells, U-87 MG cells also exhibited a significant green monomeric signal under serum-depletion conditions, indicating elevated ROS levels (Figure 12H). The addition of FBS to the culture medium restored membrane potential, as indicated by the appearance of red colored J-aggregates in these cells.

### 3.9. FBS-mediated mitochondrial protection in C6 and U-87 MG cells

The results of FBS deprivation and supplementation clearly demonstrated that the addition of FBS could restore the damage caused by its deprivation. Hence, we investigated whether this restorative effect is influenced by the glucose concentration in the culture medium. Experimentally, C6 and U-87 MG cells were cultured in a complete medium in 30 mm plates for 24 h. Subsequently, the cells were starved of glucose and FBS overnight. After 12 h of starvation, the cells were cultured in a glucose-free medium with 10% FBS, as well as in media supplemented with increasing concentrations of glucose (100 mg/dL and 400 mg/dL) along with 10% FBS.

**Figure 13:**
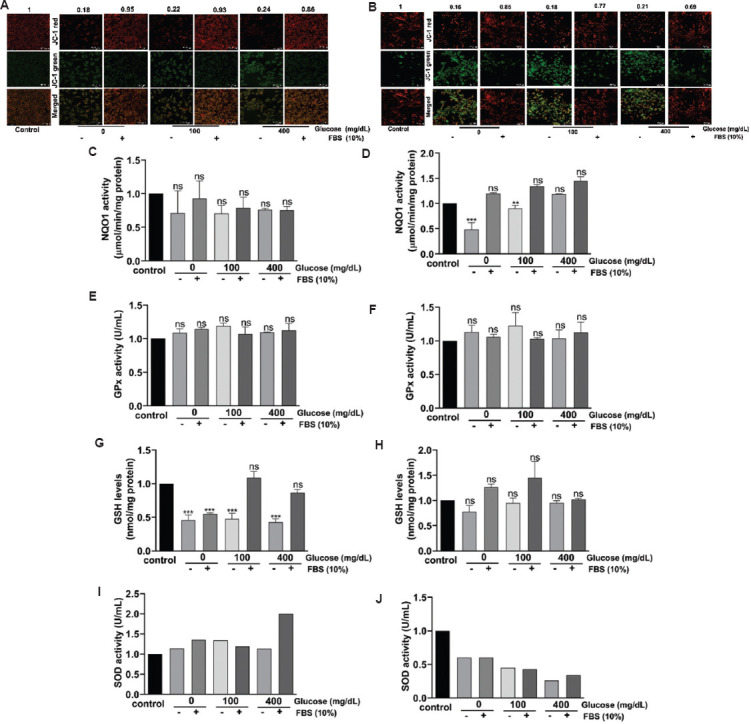
Effect of fetal bovine serum (FBS) on mitochondrial membrane potential and antioxidant enzyme levels in glioblastoma cell lines cultured in a medium containing no glucose or varied concentrations of glucose. Quantified images of (A) C6 cells and (B) U-87 MG cells treated with glucose and FBS and stained with JC-1. Red cells represent J-aggregates; green cells represent monomers. Merged images of red cells and green cells are presented in the third row. Scale bar: 72.2 μm, magnification: 20×. (C) NQO1 activity of C6 cells showed a non-significant decrease compared to cells treated with FBS (D) NQO1 activity of U-87 MG cells showed a significant decrease in activity at 24 h. (E) GSH activity of C6 cells remained high at 100 and 400 mg/dL in the presence of FBS. (F) GSH activity of U-87 MG cells showed a non-significant increase in FBS-treated glucose cells. (G) GPx activity of C6 cells remained unchanged. (H) GPx activity of U-87 MG cells at 24 h remained unchanged. (I) SOD activity in C6 cells was high at 400 mg/dL glucose. (J) SOD activity in U-87 MG cells showed no difference compared to the control. The values are presented as mean ± standard error of the means of two independent experimental values. The statistical analysis was conducted using Tukey’s *post hoc* test. Statistical significance was determined at *p<0.05, **p<0.01, and ***p<0.001. ns refers to not significant. Abbreviations: GPx: Glutathione peroxidase; GSH: Glutathione; NQO1: Nicotinamide adenine dinucleotide phosphate quinone oxidoreductase; SOD: Superoxide dismutase.

Data analysis showed that FBS addition protected cells from serum- and glucose-deprived damages, irrespective of glucose concentration in the culture medium (Figure 13A and B). Supporting these observations, analysis of the expression of antioxidant enzymes, GPX, NQO1, SOD, and intracellular GSH showed variations in their activity and quantity (Figure 13C, to J). For instance, a significant decrease in NQO1 activity was observed upon the deprivation of FBS in U-87 MG cells (Figure 13D). In contrast, in C6 cells, only a visible decrease in NQO1 activity was observed (Figure 13C). Similarly, there was a decrease in cellular GSH with FBS deprivation in U-87 MG and C6 cells (Figure 13E and F). No significant changes were observed in GPX activity after serum deprivation. SOD activity showed a moderate increase in SOD activity in C6 cells (Figure 13I). In contrast, U-87 MG showed a decrease in activity (Figure 13J). In summary, NQO1 could be seen as a marker for measuring FBS deprivation-induced effects on cells. Notably, the addition of glucose to serum-deprived cells increased the activity of NQO1 in U-87MG cells (Figure 13D).

### 3.10. FBS-induced protein kinase B phosphorylation in C6 and U-87 MG cells

**Figure 14:**
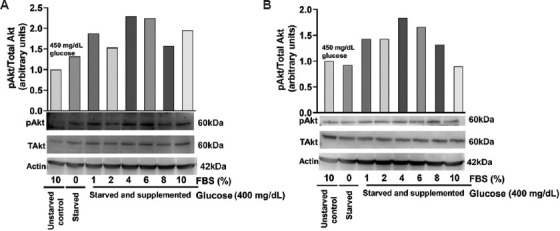
Western blot analysis of glioblastoma cell lines with different concentrations of fetal bovine serum. (A) The bar graph represents variations in pAkt levels of C6 cells compared to control cells. (B) The bar graph represents variations in the pAkt levels of U-87 MG cells compared to control cells. Abbreviations: Akt: Protein kinase B; p: Phosphorylated; T: Total.

Protein kinase B is a key mediator of insulin signaling.^28^ Upon activation through phosphorylation (at Threonine 308 and Serine 473), Akt plays a crucial role in glucose homeostasis.^29^ To investigate the effect of FBS on Akt phosphorylation, we exposed C6 and U-87 MG cells to increasing concentrations of FBS and analyzed the phosphorylation status of Akt after 24 h. Data analysis showed that FBS starvation led to an increase in Akt levels in C6 cells (Figure 14A), whereas U-87 MG cells showed no changes compared to the control (Figure 14B). However, FBS supplementation (1–10%) led to a further increase in Akt phosphorylation that was observed in both glioblastoma cells.

### 3.11. Effects of FBS deprivation on ROS levels in normal human keratinocytes

**Figure 15:**
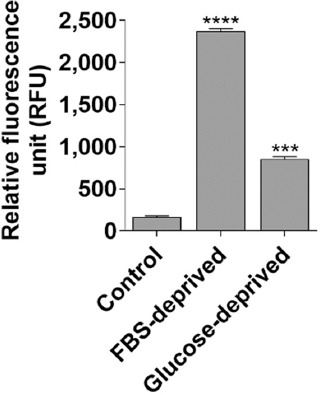
Glucose and fetal bovine serum **(F**BS) deprivation increased intracellular reactive oxygen species (ROS) levels in human keratinoc**ytes.** The representative bar graph shows changes in intracellular ROS levels at 24 h. The data represent the mean of two independent experimental values with at least three replicate wells in each experiment. The statistical analysis was conducted using Tukey’s *post* *hoc* test. Statistical significance was determined at ***p<0.001 and ****p<0.0001.

To investigate whether glucose and FBS deprivation affected normal human keratinocyte cells were cultured in complete media. After 24 h of incubation, cells were either deprived of glucose or FBS, then cultured for an additional 24 h. Data analysis revealed that both glucose and FBS deprivation significantly increased ROS levels compared to control cells. Moreover, FBS deprivation had more substantial effects on ROS levels than glucose deprivation in the human keratinocytes (Figure 15).

### 3.12. FBS-mediated neutralization of hyperglycemia-induced ROS in glioblastoma cells

**Figure 16:**
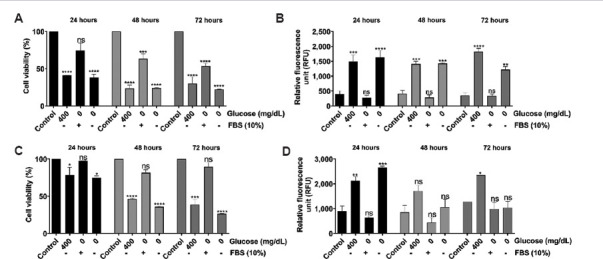
Fetal bovine serum (FBS) mediates protection against intracellular ROS levels in glioblastoma cells. (A) Percentage of cell viability of C6 cells at 24, 48, and 72 h. (B) Intracellular ROS levels of C6 cells at 24, 48, and 72 h. (C) Percentage of cell viability of U-87 MG cells at 24, 48, and 72 h. (D) Intracellular ROS levels of U-87 MG cells at 24, 48, and 72 h. The data represent the mean of two independent experimental values with at least three replicate wells in each experiment. The statistical analysis was conducted using Tukey’s *post hoc* test. Statistical significance was determined at *p<0.05, **p<0.01, ***p<0.001, and ****p<0.0001. ns refers to not significant.

Studies have shown that unusually high glucose (400 mg/dL) triggers ROS generation in cultured cells and in mice.^30^. Prior studies have reported that components in the FBS could inhibit hyperglycemia-induced ROS generation. Hence, in this study, we tested the impact of adding FBS to a culture medium. Interestingly, we found that FBS could reduce ROS levels in the glioblastoma cell lines (Figure 16).

## 4. Discussion

*In vitro* cell culture requires nutrients that are essential for cell growth and proliferation.^31^ These nutrients include glucose, glutamine, sodium pyruvate, essential amino acids, and FBS, among others. Among the supplements provided in the culture medium, glucose is considered to be a vital energy source for various cellular processes and serves as a carbon pre-cursor for the synthesis of various biomolecules.^32^ FBS, one of the most commonly used supplements in cell culture media, is required for adequate cell proliferation and growth. However, the use of FBS is highly debatable due to its animal origin.

Although prior studies have investigated the roles of glucose and FBS in cell culture, the impact of withdrawing these key components on cell viability and integrity has not been thoroughly studied, particularly in glioblastoma cell lines. Moreover, the mechanisms underlying cell death induced by glucose and FBS withdrawal remain poorly understood.^33^ Hence, in the present study, we explored the roles of glucose and FBS in glioblastoma cell lines C6 and U-87 MG by assessing their effects on ROS production, apoptosis induction, and cell cycle arrest.

We observed that glucose withdrawal differentially decreased the viability of C6 cells and U-87 MG cells. Supplementing starved cells with glucose enhanced the proliferation in a dose-dependent fashion in both cell lines. Similar to our findings, Khajah *et al*.^34^ reported that glucose withdrawal led to a lethal effect on cells and supplementation with glucose enhanced cell proliferation in a dose-dependent manner.^34^ In another study, glucose deprivation showed a delayed response to cell inhibition as the cells experienced proliferation arrest only at an extended time point.^35^ A comparable effect was also observed in our study in the human U-87 MG glioblastoma cells, wherein glucose withdrawal exerted no major effect at 24 h; however, continuation of glucose withdrawal moderately reduced cell proliferation at 48 and 72 h, suggesting that glucose deprivation affects each cell line differently.

In the *in vitro* system, the withdrawal of glucose can initiate a complex molecular response that can lead to the activation and inhibition of various signaling pathways. Earlier studies have reported an increase in tyrosine phosphorylation upon glucose starvation.^33^ Hence, in this study, we measured the phosphorylation level of Akt, a protein involved in the metabolism of glucose. We observed an increased phosphorylation of Akt upon glucose starvation in both rat and human glioblastoma cell lines. In line with our study, Gao *et al*.^36^ reported that protracted glucose deprivation induces Akt phosphorylation under metabolic stress. In addition, Graham *et al*.^33^ demonstrated that glucose deprivation induced supra-physiological levels of tyrosine phosphorylation.

Glucose withdrawal is also known to activate a positive feedback loop involving ROS.^32^ Hence, in this study, we quantitatively determined the expression and activity of GPx, SOD, and NQO1, the most widely reported antioxidant enzymes. In addition, we also quantified the concentration of GSH to determine the impact of glucose withdrawal on the cellular reducing potential. SOD and NQO1 expression decreased in a concentration-dependent manner in the rat C6 cells; however, in human U-87 MG cells, the expression of these proteins was increased. Notably, we have not observed any significant differences in the expression of GPx and the concentration of cellular GSH.

Previous studies have reported that glucose withdrawal arrests cells in the G0/G1 phase.^37^-^39^ Similar to these studies, we also found that glucose deprivation activated cell cycle arrest in the G0/G1 phase in glioblastoma cell lines. By integrating existing data and the observations from our study, we were led to conclude that glucose withdrawal plays a key role in the survival of glioblastoma cells. Although glucose is essential for cell proliferation and growth, the response of glioblastoma cells to elevated glucose levels in the medium varied according to cell lines. Therefore, it is important to study the impact of glucose on each single cell line before drawing any definite conclusions.

Serum starvation is another commonly used method to synchronize cells and evaluate the effects of growth factor-induced/inhibited signaling pathways. Understanding how glioblastoma cells respond to serum deprivation is essential. Thus, we examined the effect of serum starvation on the viability of C6 and U-87 MG cells and observed a reduction in cell viability in serum-deprived cells. A prior study by Rashid and Coombs^40^ also showed that deprivation of serum reduced the viability of human lung epithelial cells (A549).^7^,^40^,^41^ We found that the decreased viability could be mechanistically ascribed to the induced cell death, as evidenced by ethidium bromide incorporation into fragmented DNA. Similar to our findings, White *et al*.^10^ and Rashid and Coombs^40^ also reported serum deprivation-induced apoptosis in prostate cancer cells and lung A549 cancer cells, respectively.

Serum starvation plays a crucial role in cancer cell metabolism; therefore, we further investigated cellular stress by measuring ROS levels. We observed that serum deprivation triggered cellular ROS generation in both glioblastoma cells. Supplementation of serum to serum-deprived cells showed a dose-dependent decrease in cellular ROS levels. Similar to our findings, Lee *et al*.^42^ also reported that serum deprivation triggers ROS generation. The mechanism by which serum deprivation induces ROS production remains unclear. Given that cellular oxidative stress is largely regulated by mitochondrial metabolism, we examined the mitochondrial membrane potential in these cells. Our data suggest that the observed ROS generation is attributable to increased mitochondrial damage. This interpretation aligns with previous studies,^43^-^45^ which documented mitochondrial dysfunction as a contributor to serum deprivation-induced oxidative stress.

Furthermore, to explore the signaling mechanisms triggered by serum deprivation and supplementation, the serum-deprived cells were cultured in a medium supplemented with increasing concentrations of FBS. Cell lysates were then analyzed by Western blotting to assess changes in the phosphorylation status of Akt. Akt is a key protein involved in the regulation of cell proliferation and survival.^46^ Akt is known to activate nuclear factor erythroid 2-related factor 2, a key transcription factor in cellular antioxidant defense mechanisms. Cells cultured in medium containing FBS exhibited increased phosphorylation of Akt in C6 glioblastoma cells; however, there existed no significant difference in U-87 MG cells. A similar difference was also observed in the expression of antioxidant enzymes (SOD, NQO1, and GPx) between these cell lines. This different response to serum deprivation could be attributed to their distinct cellular origins and intrinsic biological differences.^47^,^48^ In summary, our study revealed that FBS deprivation in the medium induced ROS generation in glioblastoma cells, thereby promoting cell death.

Our study comprehensively explored the effects of serum and glucose deprivation on cell viability, ROS induction, and apoptosis in glioblastoma cells. Oh *et al*.^49^ reported enhanced stemness in U-87 MG glioblastoma cells, as indicated by increased expression of the natural killer group 2 member D ligand. Similarly, Gaelzer *et al*.^50^ showed that hypoxia and reoxygenation conditions in C6 glioma cells led to the dedifferentiation of cancer cells, evidenced by increased expression of Nestin and CD133. Despite the significant findings of our study, there are several limitations. A notable limitation is that we did not study the impact of serum deprivation on stemness markers and differentiation. This was primarily due to our use of long-term cultured cells, which generally do not exhibit stem-like characteristics, and the study’s focus on metabolic and oxidative stress responses as well as survival mechanisms.

Another important limitation is the extent to which our findings translate to *in vivo* conditions. Although our study provides insights into long-term cultured glioblastoma cell lines, these models may not fully reflect *in vivo* conditions, which are influenced by tumor heterogeneity and the tumor microenvironment. Hence, patient-derived cultures would serve as better models to more accurately understand the effects of serum and glucose deprivation. Furthermore, to draw significant conclusions regarding the effects of serum and glucose deprivation, similar experiments should be conducted across multiple cell lines.

Despite these limitations, our study provides valuable insights into the metabolic stress responses and survival mechanisms of glioblastoma cells under nutrient deprivation. However, further studies employing diverse cellular models, particularly patient-derived cells, are necessary to address these limitations and strengthen the drawn conclusions for cancer research.

## 5. Conclusion

Results from our study demonstrated the importance of identifying the appropriate concentration of FBS and glucose for each cell line. While the impact of glucose deprivation was observed only during extended starvation in a cell line-specific manner, serum starvation caused oxidative stress and G0/G1 arrest in both cell lines. These cell line-specific responses were observed due to changes in activities and expressions of antioxidant enzymes, intracellular ROS, and mitochondrial membrane potential differences. In summary, the cells exhibit differential responses to nutrient deprivation by altering the complex interactions within cellular networks. Therefore, future studies should account for these nutrient variables when assessing their impact on cell survival and the underlying regulatory mechanisms.

## Figures and Tables

**Figure R1:**
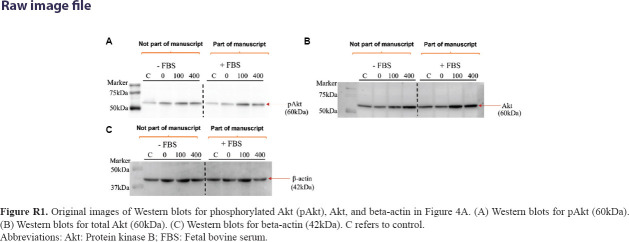
Original images of Western blots for phosphorylated Akt (pAkt), Akt, and beta-actin in Figure 4A. (A) Western blots for pAkt (60kDa). (B) Western blots for total Akt (60kDa). (C) Western blots for beta-actin (42kDa). C refers to control. Abbreviations: Akt: Protein kinase B; FBS: Fetal bovine serum.

**Figure R2:**
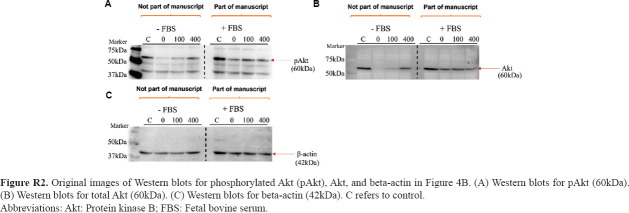
Original images of Western blots for phosphorylated Akt (pAkt), Akt, and beta-actin in Figure 4B. (A) Western blots for pAkt (60kDa). (B) Western blots for total Akt (60kDa). (C) Western blots for beta-actin (42kDa). C refers to control. Abbreviations: Akt: Protein kinase B; FBS: Fetal bovine serum.

**Figure R3:**
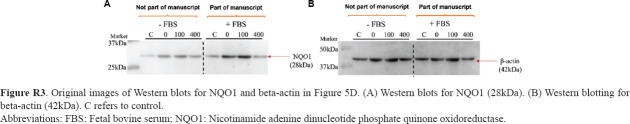
Original images of Western blots for NQO1 and beta-actin in Figure 5D. (A) Western blots for NQO1 (28kDa). (B) Western blotting for beta-actin (42kDa). C refers to control. Abbreviations: FBS: Fetal bovine serum; NQO1: Nicotinamide adenine dinucleotide phosphate quinone oxidoreductase.

**Figure R4:**
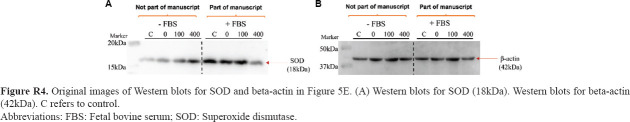
Original images of Western blots for SOD and beta-actin in Figure 5E. (A) Western blots for SOD (18kDa). Western blots for beta-actin (42kDa). C refers to control. Abbreviations: FBS: Fetal bovine serum; SOD: Superoxide dismutase.

**Figure R5:**
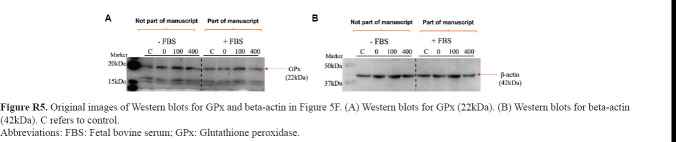
Original images of Western blots for GPx and beta-actin in Figure 5F. (A) Western blots for GPx (22kDa). (B) Western blots for beta-actin (42kDa). C refers to control. Abbreviations: FBS: Fetal bovine serum; GPx: Glutathione peroxidase.

**Figure R6:**
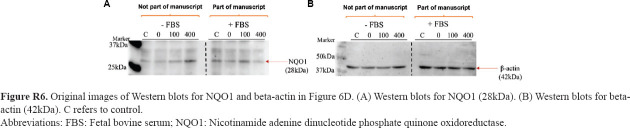
Original images of Western blots for NQO1 and beta-actin in Figure 6D. (A) Western blots for NQO1 (28kDa). (B) Western blots for beta-actin (42kDa). C refers to control. Abbreviations: FBS: Fetal bovine serum; NQO1: Nicotinamide adenine dinucleotide phosphate quinone oxidoreductase.

**Figure R7:**
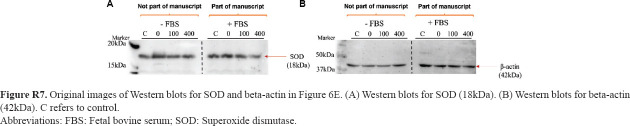
Original images of Western blots for SOD and beta-actin in Figure 6E. (A) Western blots for SOD (18kDa). (B) Western blots for beta-actin (42kDa). C refers to control. Abbreviations: FBS: Fetal bovine serum; SOD: Superoxide dismutase.

**Figure R8:**
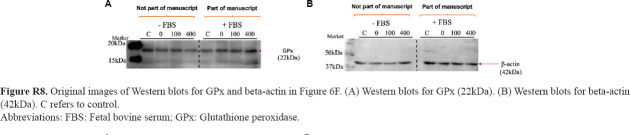
Original images of Western blots for GPx and beta-actin in Figure 6F. (A) Western blots for GPx (22kDa). (B) Western blots for beta-actin (42kDa). C refers to control. Abbreviations: FBS: Fetal bovine serum; GPx: Glutathione peroxidase.

**Figure R9:**
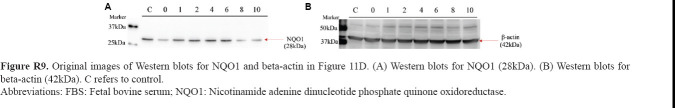
Original images of Western blots for NQO1 and beta-actin in Figure 11D. (A) Western blots for NQO1 (28kDa). (B) Western blots for beta-actin (42kDa). C refers to control. Abbreviations: FBS: Fetal bovine serum; NQO1: Nicotinamide adenine dinucleotide phosphate quinone oxidoreductase.

**Figure R10:**
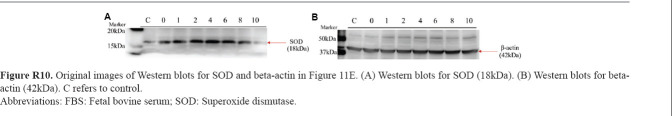
Original images of Western blots for SOD and beta-actin in Figure 11E. (A) Western blots for SOD (18kDa). (B) Western blots for beta-actin (42kDa). C refers to control. Abbreviations: FBS: Fetal bovine serum; SOD: Superoxide dismutase.

**Figure R11:**
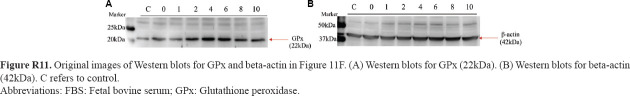
Original images of Western blots for GPx and beta-actin in Figure 11F. (A) Western blots for GPx (22kDa). (B) Western blots for beta-actin (42kDa). C refers to control. Abbreviations: FBS: Fetal bovine serum; GPx: Glutathione peroxidase.

**Figure R12:**
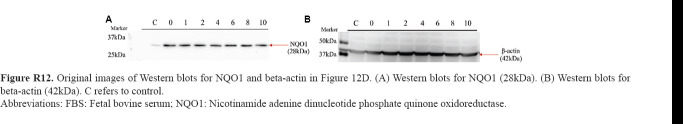
Original images of Western blots for NQO1 and beta-actin in Figure 12D. (A) Western blots for NQO1 (28kDa). (B) Western blots for beta-actin (42kDa). C refers to control. Abbreviations: FBS: Fetal bovine serum; NQO1: Nicotinamide adenine dinucleotide phosphate quinone oxidoreductase.

**Figure R13:**
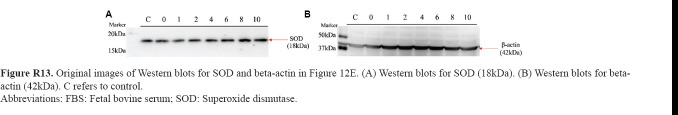
Original images of Western blots for SOD and beta-actin in Figure 12E. (A) Western blots for SOD (18kDa). (B) Western blots for beta-actin (42kDa). C refers to control. Abbreviations: FBS: Fetal bovine serum; SOD: Superoxide dismutase.

**Figure R14:**
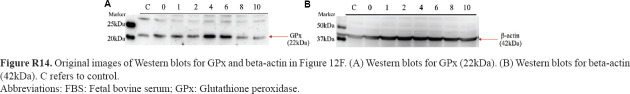
Original images of Western blots for GPx and beta-actin in Figure 12F. (A) Western blots for GPx (22kDa). (B) Western blots for beta-actin (42kDa). C refers to control. Abbreviations: FBS: Fetal bovine serum; GPx: Glutathione peroxidase.

**Figure R15:**
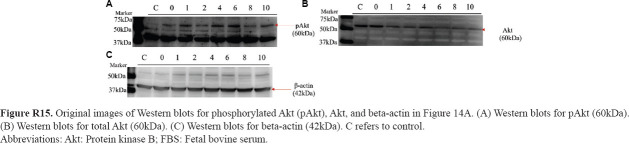
Original images of Western blots for phosphorylated Akt (pAkt), Akt, and beta-actin in Figure 14A. (A) Western blots for pAkt (60kDa). (B) Western blots for total Akt (60kDa). (C) Western blots for beta-actin (42kDa). C refers to control. Abbreviations: Akt: Protein kinase B; FBS: Fetal bovine serum.

**Figure R16:**
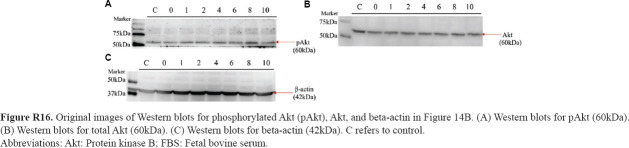
Original images of Western blots for phosphorylated Akt (pAkt), Akt, and beta-actin in Figure 14B. (A) Western blots for pAkt (60kDa). (B) Western blots for total Akt (60kDa). (C) Western blots for beta-actin (42kDa). C refers to control. Abbreviations: Akt: Protein kinase B; FBS: Fetal bovine serum.

## Data Availability

All data analyzed have been presented in the paper.
